# Anodic Stripping Voltammetric Procedure of Thallium(I) Determination by Means of a Bismuth-Plated Gold-Based Microelectrode Array

**DOI:** 10.3390/s24041206

**Published:** 2024-02-13

**Authors:** Mieczyslaw Korolczuk, Mateusz Ochab, Iwona Gęca

**Affiliations:** Institute of Chemical Sciences, Faculty of Chemistry, Maria Curie Sklodowska University, 20-031 Lublin, Poland; mieczyslaw.korolczuk@mail.umcs.pl (M.K.); mateusz.ochab@mail.umcs.pl (M.O.)

**Keywords:** thallium, array of gold microelectrodes, reusability, anodic stripping voltammetry, determination

## Abstract

This article presents a new working electrode based on a bismuth-plated, gold-based microelectrode array, which is suitable for determining thallium(I) species using anodic stripping voltammetry (ASV). It allowed a significant increase in the sensitivity as compared to other voltammetric sensors. The main experimental conditions and the instrumental parameters were optimized. A very good proportionality between the Tl(I) peak current and its concentration was evidenced in the range from 5 × 10^−10^ up to 5 × 10^−7^ mol L^−1^ (R = 0.9989) for 120 s of deposition and from 2 × 10^−10^ up to 2 × 10^−7^ mol L^−1^ (R = 0.9988) for 180 s. A limit of detection (LOD) of 8 × 10^−11^ mol L^−1^ for a deposition time of 180 s was calculated. The effects of interfering ions on the Tl(I) analytical signal were studied. The proposed method was applied for quantitative Tl(I) detection in water certified reference material TM 25.5 as well as in spiked real water samples, for which satisfactory recovery values between 98.7 and 101.8% were determined.

## 1. Introduction

The use of microelectrode arrays attracted an increased interest due to their benefits [1]. Generally, they exhibit small capacitive charging currents, reduced ohmic drop, and steady-state diffusion currents [2]. Various design arrangements of microelectrodes arrays are reported in the literature, such as arrays of (i) microdisc electrodes, (ii) microband electrodes, (iii) interdigitated microelectrodes, and (iv) linear and three-dimensional microelectrodes [3]. In addition, different techniques for building the microelectrode arrays are available [4], including photolithography [5], electrodeposition [6,7], modification [8], screen-printed techniques [9,10,11], and laser patterning [12,13].

The validity of thallium monitoring in the environment results from the fact that it is an extremely toxic element for animal organisms [14,15,16]. Thallium salts are a very potent, accumulating poison. An intake of 15 mg of dissolved thallium salts per kilogram of body weight is extremely hazardous for human health. As thallium is absorbed through the skin, mucous membranes, and lungs, poisoning caused by this element is, therefore, possible through inhalation, ingestion, and skin contact as well. Thallium accumulates in many internal organs such as the kidneys, bones, stomach, intestines, spleen, liver, muscles, lungs, and brain. The routes by which it is delivered to the organism are not important, as it behaves as a potassium analogue and is, therefore, distributed throughout the body. Thallium is a poison at the cellular level as it damages ribosomes, thereby affecting the synthesis of proteins. The described element inhibits both the vital process of cellular respiration and the activity of many enzymes, negatively affecting essential metabolic and synthesis processes. These data indicate the high importance of thallium ion monitoring in various types of environmental samples.

Various analytical methods have been proposed to determine thallium ions. The most common spectroscopic techniques have been used, such as atomic absorption spectrometry ET–AAS [17,18,19], HG–AAS [20], graphite furnace atomic absorption spectrometry GF–AAS [21], atomic emission spectrometry ICP–OES [22,23,24], and a very sensitive inductively coupled plasma mass spectrometry ICP–MS [25,26].

In the course of thallium(I) determination, stripping voltammetry is often chosen as an alternative to spectroscopic methods because of relatively inexpensive and portable equipment, a short time of measurement, and the possibility of obtaining low detection and quantification limits. Most commonly, mercury or bismuth have been used in various constructions and modifications as a working electrode material for Tl(I) determinations via anodic stripping voltammetry. The following have been used so far: hanging mercury drop electrodes [27], Nafion/mercury-film-modified electrodes [28], mercury film electrodes [29], bismuth film electrodes [30,31,32], bismuth bulk annular band electrodes [33], surface-modified thick-film graphite electrodes with Bi nanopowder [34], integrated three-electrode screen-printed sensor-modified electrodes with bismuth film [35,36], and solid bismuth microelectrodes [37]. Other solutions and electrode modifications have also been reported [38,39,40,41,42,43,44,45,46,47]. Among them, graphite microelectrodes, nano-sized ionic imprinted polymers, SnO_2_@MWCNTs-modified glassy carbon electrodes, antimony film electrodes, Nafion-coated bismuth film, Nafion-coated mercury film electrodes, ionic liquid/graphene modified electrodes, chemically modified electrodes with Langmuir–Blodgett film of p-allylcalix[4]arene, a carbon-paste electrode modified with a crown ether, amino acid functionalized glassy carbon electrodes, and an 8-hydroxyquinoline-modified carbon-paste electrode have been described. The procedures of thallium determination ought to be highly sensitive due to thallium’s extreme toxicity and rare occurrence in the environment. To the best of our knowledge, the voltammetric procedures described in [31,33,35] are characterized by the lowest values of detection limits. However, in [31], a more advanced arrangement was used, consisting of two working electrodes in one electrochemical cell. In [33], a high sensitivity was achieved at a relatively high preconcentration time, while in [35], an integrated three-electrode screen-printed sensor with a short application time was used. This is why the development of very sensitive voltammetric procedures for thallium(I) determination is still highly recommended.

Recently, a new type of gold microelectrode array with an extended lifetime, due to the way it is designed, has been proposed [48]. Gold is a popular and frequently used electrode material due to the following benefits it provides: fast electron transfer kinetics, high conductivity, its potential window with a relatively wide anodic range, reduced risk of corrosion resulting from a low reactivity in the supporting electrolyte, and its environmentally friendly character. In the present study, the amount of gold used for microelectrode array fabrication was relatively low in comparison to the conventionally sized electrodes, and it was equal to about 10 mg. This fact makes the proposed fabrication of microelectrode arrays relatively inexpensive. Thanks to the way it is construction, the electrode is easy to use and does not require any surface modifications, which, in turn, leads to excellent repeatability and reproducibility of the obtained measurement results.

The present article demonstrates the use of a new voltammetric sensor based on a reusable gold microelectrode array for thallium ion quantification via anodic stripping voltammetry. The solid gold microelectrode array reported for the first time in [48] was modified with bismuth film to meet some specific requirements regarding thallium(I) determinations. During the investigations described below, it was observed that the presented microelectrode array is an excellent substrate for metal film pre-plating. It was found that the developed combination of preparation of a working electrode leads to obtaining a very satisfactory analytical performance for thallium ion determination. The obtained detection limit was equal to 8 × 10^−11^ mol L^−1^ (deposition time 180 s). The results of studies on interference effects led to the conclusion that the proposed procedure is highly selective. The results of the analysis of the certified reference material confirm the correctness of the described analytical procedure. The obtained results of recovery studies in the course of real water samples analysis confirm the possibility of utilizing the developed procedure for Tl(I) determination in real water samples.

## 2. Materials and Methods

### 2.1. Instrumentation

The voltammetric measurements were conducted with the µAutolab (from ECO/Chemie, Utrecht, The Netherlands) in a three-electrode system containing the array of gold microelectrodes as a working electrode, a platinum wire as a counter electrode, and a Ag/AgCl/NaCl reference electrode. The electrochemical cell had a volume of 10 mL. A detailed description of the construction of an array of gold microelectrodes was given in [48]. Briefly, for fabrication of the array of gold microelectrodes, a homemade silica preform containing 792 holes was used. The outer diameter of a preform was equal to 3 mm. The holes have a nearly equilateral triangle shape with a side of about 18 µm. The minimal distance between holes was 48 µm. The holes in the silica preform were filled with melted gold using a quasi-similar procedure to that reported in [49,50,51]. In short, gold melted at a temperature of about 1140 °C was pressed in the holes of the preform under a pressure of 20 bars. After that, 5 mm preform was polished at its both ends and placed in the PEEK housing of a diameter of 6 mm. Graphitized carbon black powder and a copper wire were used for the preparation of an electrical contact from gold microelectrodes in the array. In such a way. a high-stability, long-term use sensor was obtained that can be used for at least three years without observing any significant changes in the obtained analytical signals. The surface of the microelectrodes array was polished once a day directly before the measurements with a sandpaper of 2500 grit, then it was rinsed in deionized water and kept in an ultrasonic bath for thirty seconds. A real view of the gold microelectrode array was taken by MA200 Inverted Metallographic Microscope Nikon (Tokyo, Japan).

### 2.2. Reagents

All used chemical reagents were of analytical reagent grade or Suprapur. Deionized water obtained from Milli-Q system purification was used for the preparation of all solutions. Next, 1 mol L^−1^ acetate buffer of pH of 5.3 was prepared from CH_3_COOH and NaOH, Suprapur reagents purchased from Merck. The acetate buffer solution was prepared as follows: 57.1 cm^3^ of 17.5 mol L^−1^ CH_3_COOH was added to about 800 mL of deionized water. Then 10 mol L^−1^ NaOH was added to give a post-mixing pH value of 5.30. Finally, water was added to a final volume of 1 L. A stock Tl(I) nitrate solution (concentration of 1 g L^−1^) was obtained from Fluka (Buchs, Switzerland). The working solutions of Tl(I) at concentrations of 1 × 10^−5^ or 1 × 10^−6^ mol L^−1^ were prepared by dilution of the stock solution in 0.01 mol L^−1^ HNO_3_ as required. Then, 0.2 mol L^−1^ Na_2_EDTA was obtained by appropriate dissolution of the reagent (purchased from Sigma-Aldrich, St. Louis, USA) in deionized water. A standard Bi(III) nitrate solution at a concentration of 1 g L^−1^ was purchased from Fluka. A working solution of Bi(III) at a concentration of 100 mg L^−1^ was obtained by appropriate dilution of the stock solution in 5% HNO_3_. For the procedure validation, the certified reference material TM-25.5 (from Environment and Climate Change, Canada) was used.

### 2.3. Preparation of Real Water Sample

A real water sample collected from Bystrzyca River was filtered with a 0.45 µm membrane filter, acidified to pH of 1.5 by appropriate addition of 1 mol L^−1^ HNO_3,_ and mineralized by UV irradiation for 4 h. After that, the sample was stored in polypropylene bottles at a temperature of 4 °C before a proper analysis.

A Lake Ontario water certified reference material was mineralized within 4 h using a UV irradiator, however, it was not acidified before mineralization as the mentioned material was stabilized with 0.2% HNO_3_. The pH was neutralized with an appropriate addition of 1 mol L^−1^ NaOH. The mineralization time used in presented studies is typical for this process in the case of environmental water samples [52,53].

### 2.4. Standard Procedure of Measurements

The analyzed sample was placed into the measuring cell and then 0.5 mL 1 mol L^−1^ of acetate buffer (pH 5.3), 42 μL 100 mg L^−1^ Bi(III), and 100 μL 0.2 mol L of Na_2_EDTA (used for interfering ions complexation) were added and made up to 10 mL with deionized water. The potential sequence applied to the working electrode was changed as follows: firstly, cleaning of the microelectrode’s surface was conducted at the potential of 0.5 V within 10 s; then simultaneous deposition of bismuth and thallium was conducted at −1.25 V within 120 s; then after a ten-second equilibration step, a square wave voltammogram was recorded with the changing potential from −1.1 to 0.5 V. The square wave parameters were as follows: frequency: 400 Hz, amplitude: 25 mV, and step potential: 6 mV. The measurements were carried out from non-deoxygenated solutions. Unless otherwise stated, studies were conducted at thallium(I) concentration of 2 × 10^−8^ mol L^−1^.

## 3. Results

**Figure 1:**
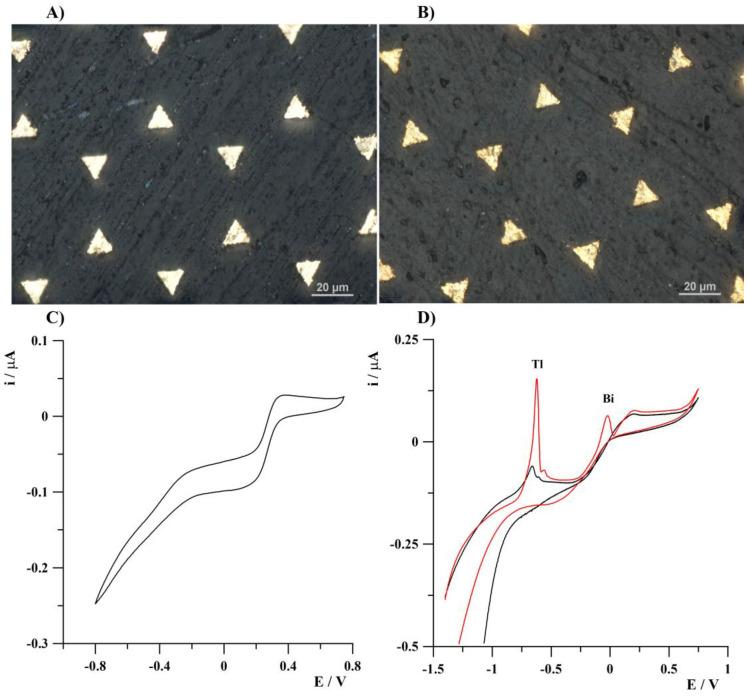
An optical image of a fragment of the surface of a gold microelectrode array (**A**); a bismuth-plated gold-based microelectrode array (**B**); cyclic voltammogram obtained from a solution containing 1 × 10^−3^ mol L^−1^ K_3_Fe(CN)_6_ and 1 mol L^−1^ KCl at scan rate of 20 mV s^−1^ (**C**); cyclic voltammograms obtained using gold microelectrodes array at scan rate of 100 mV s^−1^ from a solution containing 1 × 10^−6^ mol L^−1^ Tl(I) (black line) or 1 × 10^−6^ mol L^−1^ Tl(I) and 5 × 10^−6^ mol L^−1^ Bi(III) (red line) (**D**). Deposition conditions: −1.25 V, 120 s. Bismuth was plated in situ according to the conditions described in the standard procedure of measurements section from a solution containing Bi(III) at a concentration of 2 × 10^−6^ mol L^−1^ (B) or 5 × 10^−6^ mol L^−1^ (**D**).

Preliminary experiments show that for the preconcentration and determination of thallium(I) by anodic stripping voltammetry, a bismuth-plated gold-based microelectrode array can be successfully utilized. The mentioned solid gold microelectrode array was previously used in the case of selenium(IV) determination without any metal plating steps [48]. In the studies presented below, the use of this microelectrode array as a substrate for a bismuth film was described. Figure 1A shows the optical image of a fragment of an unmodified surface of a gold microelectrode array and Figure 1B illustrates a fragment of the surface of a gold microelectrode array electrochemically modified with a bismuth film. It could be observed that the surface of the unmodified gold microelectrode array is more reflective and shiny.

To assess the interaction between diffusion layers of individual microelectrodes, a cyclic voltammogram was recorded from a solution containing 1 × 10^−3^ mol L^−1^ K_3_Fe(CN)_6_ and 1 mol L^−1^ KCl at a scan rate of 20 mV s^−1^. The obtained results are shown in Figure 1C. These results and the literature data [54] indicate a mixed diffusion layer over the gold microelectrode array because of partial overlapping of particular diffusion layers.

The approach of using a bismuth-plated gold-based microelectrode array results in a significant increase in the efficiency of thallium deposition that was confirmed by cyclic voltammograms recorded from a solution containing 1 × 10^−6^ mol L^−1^ Tl(I) in the presence or absence of 5 × 10^−6^ mol L^−1^ Bi(III). The obtained results presented in Figure 1D show a significant increase in thallium analytical signal in the presence of Bi(III). The next important benefit of bismuth film plating is the extension of the potential window in the cathodic range from about −0.9 V to −1.1 V. The above-mentioned facts confirm the validity of the modification of the surface of the array of microelectrodes with a bismuth film.

Further advantages of this solution such as a very low detection limit, a very good repeatability of the obtained results, and a high selectivity, as well as the development of voltammetric procedure of Tl(I) determination, are described as follows.

### 3.1. Optimization of the Acetate Buffer, Bi(III), and Na_2_EDTA Concentration and a pH of Acetate Buffer

**Figure 2:**
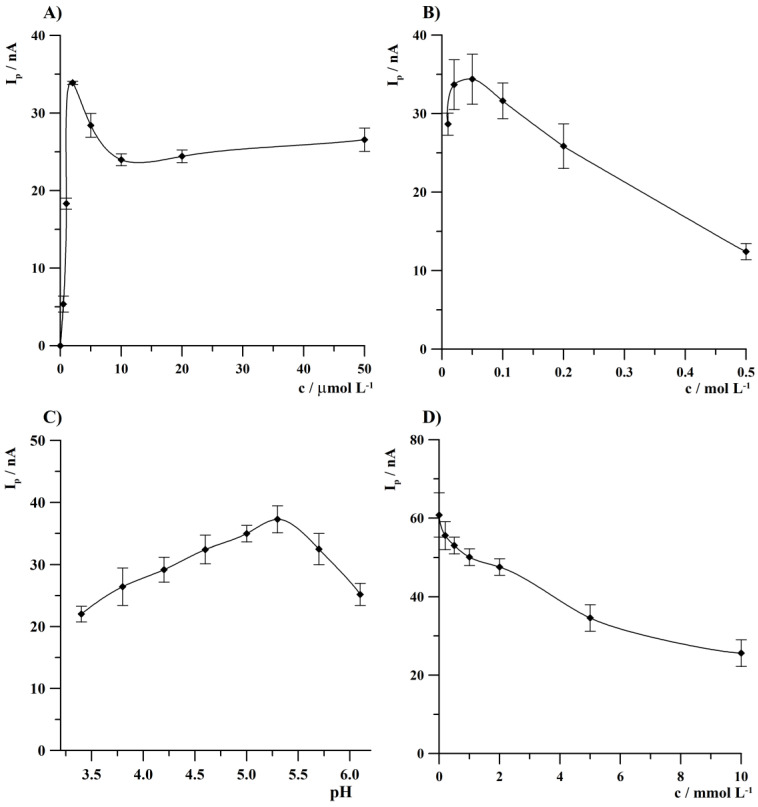
The effect of Bi(III) concentration (**A**); buffer concentration (**B**); pH of acetate buffer (**C**); and Na_2_EDTA concentration (**D**) on thallium peak current. Tl(I) concentration was 2 × 10^−8^ mol L^−1^. Deposition conditions: −1.35 V, 120 s.

Preliminary experiments performed using an array of gold microelectrodes led to the conclusion that the addition of Bi(III) ions to the supporting electrolyte is necessary in case of anodic stripping voltammetric thallium determination, as in the absence of Bi(III), no Tl(I) peak current was observed for low concentrations of the latter. The effect of Bi(III) concentration on Tl(I) peak height was investigated within the range from 5 × 10^−7^ to 5 × 10^−5^ mol L^−1^. Tl(I) and Na_2_EDTA concentration was 2 × 10^−8^ and 5 × 10^−3^ mol L^−1^, respectively. The obtained results are shown in Figure 2A. As it was observed, the current of the Tl(I) peak increased with an increase in the Bi(III) ion concentration from 0 to 2 × 10^−6^ mol L^−1^ and then decreased at higher bismuth ions concentrations. For low Bi(III) ions concentrations, deposition of Tl rapidly increases as a result of a previously described co-deposition effect of bismuth and other metals determined by anodic stripping voltammetry [55]. The decrease in the Tl(I) analytical signal at higher Bi(III) concentration can be explained by a multilayer formation of bismuth that was reported previously in [30]. On the basis of these results, the concentration of Bi(III) equal to 2 × 10^−6^ mol L^−1^ was considered to be the optimal value.

The concentration of acetate buffer (pH 4.6) chosen as a supporting electrolyte was studied from 0.01 to 0.5 mol L^−1^. The obtained results presented in Figure 2B indicate that the Tl(I) peak current attained the highest value at the concentration of acetate buffer of 0.05 mol L^−1^ and then the thallium signal decreased with higher buffer concentrations.

The impact of pH of acetate buffer was studied from 3.4 to 6.1. The experiment results are shown in Figure 2C. It was observed that Tl(I) peak height was growing up to a pH of 5.3. At higher pH values, a decrease in Tl(I) voltammetric response was observed. This observation can be explained by the fact that Bi(III) is hydrolyzed at higher pH values with the formation of a precipitate that results in a decrease in the concentration of dissolved Bi(III). As a result, a favorable concentration and pH values of acetate buffer 0.05 mol L^−1^ and 5.3 were selected, respectively.

The next optimized parameter was a concentration of Na_2_EDTA. The presence of this complexing agent in the analyzed solution enables the determination of thallium(I) in the presence of multivalent ions, as it results in the complexation of the latter into electrochemically inactive forms at the potential of thallium deposition, thereby increasing the selectivity of the analytical procedures of the determination of monovalent ions. This fact was plainly explained and illustrated in [56]. The impact of Na_2_EDTA concentration on T(I) peak height was tested from 0 to 0.01 mol L^−1^. The results of this study are shown in Figure 2D. It was found that the highest thallium signal was obtained in the absence of Na_2_EDTA and then it decreased with increasing concentration of the complexing agent. Because the addition of Na_2_EDTA to the supporting electrolyte is crucial in the case of Tl(I) determination in the presence of polyvalent ions, the Na_2_EDTA concentration of 2 × 10^−3^ mol L^−1^ was selected for further studies as a compromise between the complexing agent concentration and a drop of Tl(I) analytical signal. At the lowest tested Na_2_EDTA concentrations, an increase in interference effects from polyvalent ions may be observed, however, the highest studied concentration of this complexing agent caused a decrease in the thallium signal to about 50%.

### 3.2. Square Wave Parameters Optimization

Because main square wave parameters (frequency, amplitude, step potential) may affect the peak current of analytes, their optimization was carried out during the procedure development.

**Figure 3:**
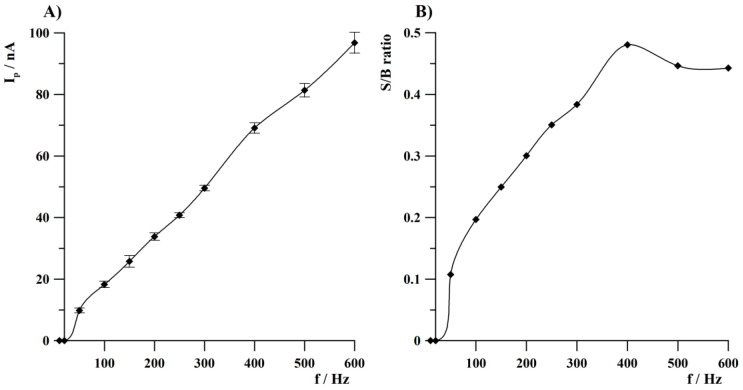
The effect of frequency on thallium peak current (**A**) and signal-to-background ratio as a function of frequency (**B**). Tl(I) concentration was 2 × 10^−8^ mol L^−1^. Deposition conditions: −1.35 V, 120 s.

The effect of frequency on Tl(I) peak current was investigated from 10 to 600 Hz. Amplitude and step potential were 15 and 4 mV, respectively. It was observed that Tl(I) peak height increased in the whole range of tested values from 10 to 600 Hz, which is presented in Figure 3A. Moreover, signal-to-background ratios (S/B) were calculated and their dependence as a function of frequency is presented in Figure 3B. The increase in S/B ratios at an array of gold microelectrodes was observed up to 400 Hz and then slightly decreased at higher frequency values (about 90% of a maximum value of Tl(I) peak current). Based on the described results, further studies were conducted at a frequency of 400 Hz. The enhanced S/B ratio observed on an array of gold microelectrodes is an important feature that may lead to an increase in the sensitivity of voltammetric determinations.

The effect of amplitude on Tl(I) voltammetric response was examined from 10 to 100 mV while frequency and step potential were 400 Hz and 4 mV, respectively. The Tl(I) signal increased with increasing amplitude values up to 25 mV, reached the greatest peak height within the range from 25 to 50 mV and then decreased with increasing values of amplitude. The amplitude value of 25 mV was taken for further studies.

The effect of step potential on Tl(I) peak current was studied from 2 to 12 mV while frequency and amplitude were 400 Hz and 25 mV, respectively. Tl(I) analytical response increased in the whole range of tested values, however, a significant signal increase was observed from 2 to 6 mV, and then, at higher values of step potential, a slower enhancement of Tl(I) signal was detected in the range from 6 to 12 mV. The most favorable S/B ratio was obtained at a step potential of 6 mV and this value was chosen for further research.

### 3.3. Optimization of the Measurement Conditions

**Figure 4:**
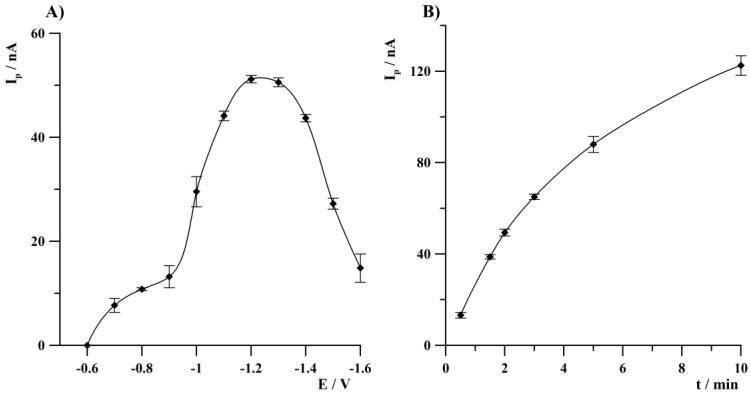
The influence of deposition potential (**A**) and deposition time (**B**) on thallium peak height. Tl(I) concentration: 1 × 10^−8^ mol L^−1^. Deposition conditions: 120 s (**A**); −1.25 V (**B**). Frequency, amplitude, and step potential were 400 Hz, 25, and 6 mV, respectively.

Another parameter requiring optimization that affected Tl(I) peak height was deposition potential. Within the deposition step, simultaneous preconcentration of bismuth and thallium was conducted, which led to a shortened total measurement time. The effect of a mentioned parameter on Tl(I) peak height was studied from −0.6 to −1.6 V and the obtained results are illustrated in Figure 4A. Deposition time was 120 s. The concentration of Tl(I) was equal to 1 × 10^−8^ mol L^−1^. The Tl(I) peak height increased from −0.6 V, obtaining the highest value within the range from −1.2 to −1.3 V and then decreased at more negative values of deposition potential. Further studies were carried out at a deposition potential of −1.25 V.

The influence of deposition time was tested from 30 to 600 s. During this investigation, Tl(I) concentration was 1 × 10^−8^ mol L^−1^. The results are presented in Figure 4B. The Tl(I) peak current increased almost linearly up to 300 s and then also increased but more slowly to 600 s of deposition. Further research were conducted at 120 s of deposition to shorten the total measurement time but for determination of the lowest Tl(I) concentrations, a longer deposition time is recommended. Sensitivity of determinations can be enhanced by extending the deposition time.

### 3.4. Calibration Studies

**Figure 5:**
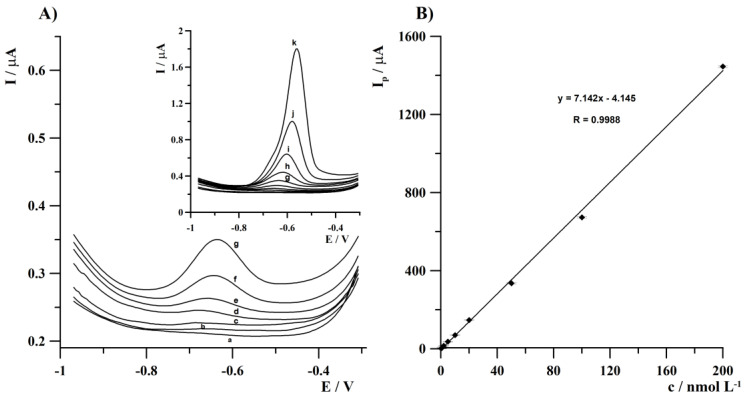
(**A**) Anodic stripping voltammograms obtained for increasing Tl(I) concentrations for 180 s of deposition: (a) blank; (b) 2 × 10^−10^; (c) 5 × 10^−10^; (d) 1 × 10^−9^; (e) 2 × 10^−9^; (f) 5 × 10^−9^; (g) 1 × 10^−8^; (h) 2 × 10^−8^; (i) 5 × 10^−8^; (j) 1 × 10^−7^; (k) 2 × 10^−7^ mol L^−1^. Concentration and pH of acetate buffer: 0.05 mol L^−1^ and 5.3, respectively; the potential of deposition: −1.25 V; frequency, amplitude, and step potential: 400 Hz, 25, and 6 mV, respectively. (**B**) Linear calibration graph. Error bars represent relative standard deviation (n = 3).

Calibration curves were established for two deposition times. A linearity within three orders of magnitude was found in both cases described further. The dependence of Tl(I) peak current on its concentration for 120 s of deposition was a straight line within the concentration range from 5 × 10^−10^ to 5 × 10^−7^ mol L^−1^ and is described by the equation y = 3.95 x + 18.71, where y is the peak current (nA) and x is Tl(I) concentration (nmol L^−1^) (R = 0.9989). The detection limit for Tl(I) estimated as three times the standard deviation of intercept divided by the slope of the calibration plot was equal to 2.2 × 10^−10^ mol L^−1^ for 120 s of deposition. The second calibration graph was designated for 180 s of deposition. In this case, linearity was found over the concentration range from 2 × 10^−10^ to 2 × 10^−7^ mol L^−1^ and is expressed as follows: y = 7.14 x − 4.14 (R = 0.9988). The detection limit for Tl(I) was equal to 8 × 10^−11^ mol L^−1^ (for deposition time of 180 s). The estimated value of the limit of detection can be further lowered by prolongation of deposition time. The recorded voltammograms obtained during the calibration studies for 180 s of deposition are presented in Figure 5A. The analytical parameters characterizing the developed procedure for voltammetric thallium ions determination are compared in Table 1. The comparison of detection limits of the previously reported voltammetric procedures using various mercury-free, metal-working electrodes in the course of Tl(I) determination is summarized in Table 2. Based on the values presented in Table 2, it can be seen that the lowest reported LOD value was obtained in [31,33,35], however in [33], longer deposition times were applied, while in [35] both (*) integrated three-electrode screen-printed sensor with a short application time and (**) background-subtraction method were used during research. Studies described in [31] concern the application of two working electrodes during one measurement cycle thanks to which an initial preconcentration of the analyte was achieved using the first working electrode. In turn, the unquestionable advantage of the research presented in this article is the application of a new type of reusable metal microelectrode array with an extended lifetime and simplicity of a surface preparation.

### 3.5. Repeatability Studies

The repeatability of the analytical signal for the solid gold array microelectrodes modified with bismuth film used for Tl(I) ions determination was investigated. The signals obtained for nine subsequent measurements performed from the same sample containing components of the supporting electrolyte with 1 × 10^−8^ mol L^−1^ of Tl(I) were compared. Deposition time was 120 s. The calculated value of RSD was equal to 2.75%. The voltammograms obtained during the described studies presented in Figure S1 in Supplementary Material confirm the excellent repeatability of thallium(I) determinations.

### 3.6. Interferences

The interferences related to the presence of interfering ions on Tl(I) peak height were studied for ions typically observed in natural water samples. During the described research, Tl(I) concentration was equal to 1 × 10^−8^ mol L^−1^ (120 s of deposition). The obtained results indicate that 100-fold excess of a majority of tested ions did not significantly influence Tl(I) analytical signal. Only in the case of Cu(II) was a more pronounced effect on the Tl(I) signal observed—the presence of 50-fold excess of copper ions caused a decrease in thallium peak current to 59% of its original value. The more detailed data concerning the results of interference studies are summarized in Table 3.

### 3.7. Procedure Validation

**Figure 6:**
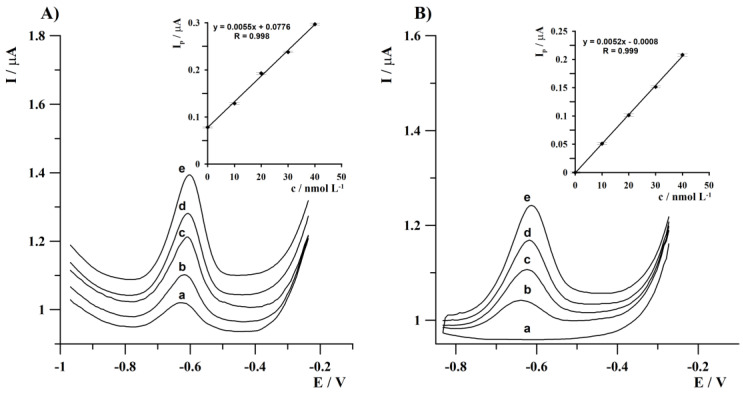
Anodic stripping voltammograms were obtained during the analysis of: (**A**) certified reference material TM 25.5; and (**B**) real water sample. (**A**): (a) sample of TM 25.5; (b) as (a) + 1 × 10^−8^; (c) as (a) + 2 × 10^−8^; (d) as (a) + 3 × 10^−8^; (e) as (a) + 4 × 10^−8^ mol L^−1^. (**B**): (a) sample of diluted river water; (b) as (a) + 1 × 10^−8^; (c) as (a) + 2 × 10^−8^; (d) as (a) + 3 × 10^−8^; (e) as (a) + 4 × 10^−8^ mol L^−1^. Deposition at −1.25 V within 120 s. Insets: standard addition graphs. Error bars represent relative standard deviation (n = 3).

The correctness of the developed procedure was verified by Tl(I) determination in natural lake water certified reference material TM 25.5 using the standard additions method at deposition time of 120 s. The result obtained after analysis of TM 25.5 (dilution factor: 10) equal to 29 µg L^−1^ with a standard deviation of 3.5% (n = 5) is consistent with the certified value of 30.0 µg L^−1^ (±) 2.8. Anodic stripping voltammograms obtained in the course of an analysis of the certified reference material TM 25.5 are shown in Figure 6A.

The next purpose of this research was a practical application of the developed procedure for the determination of Tl(I) in the environmental water samples from the Bystrzyca River (eastern part of Poland). After an appropriate preparation described in Section 2.3, 5 mL of the sample was taken to the electrochemical cell (factor of dilution: 2) and the voltammetric measurements were carried out using the standard addition method. The voltammograms recorded from the Bystrzyca River water sample did not exhibit a Tl(I) analytical signal, which indicated that the thallium concentration was below the detection limit of the described procedure. Therefore, recovery studies were performed by spiking the analyzed samples with fixed Tl(I) concentrations to confirm the accuracy of the developed procedure. Three replicate determinations of each sample containing 1 × 10^−8^ mol L^−1^ Tl(I) resulted in average recovery values in the range from 98.7 to 101.8% with a relative standard deviation (RSD) of 3.4%. The voltammograms obtained during this analysis are presented in Figure 6B. The obtained results confirmed that the developed procedure can be used for Tl(I) determination in real water samples. The results of analysis of the certified reference material and real water sample are summarized in Table 4.

## 4. Conclusions

The presented article demonstrates the utilization of a new voltammetric working electrode—a solid gold microelectrode array. The mentioned working electrode previously proposed in [48] was used for the first time as a substrate for a bismuth film and utilized for the determination of thallium ions by anodic stripping voltammetry. The results presented in these studies indicate that the mentioned array of microelectrodes is an excellent substrate for bismuth film that can be successfully applied for thallium(I) determination by anodic stripping voltammetry. The described procedure is characterized by three orders of magnitude linear dynamic range, a very low detection limit equal to 8 × 10^−11^ mol L^−1^ (for 180 s of deposition), and a very good repeatability of the obtained results (RSD 2.75%). The analytical procedure described in this article can be used for environmental monitoring for thallium(I) determination, which was confirmed by analysis of the natural certified water reference material as well as natural water samples.

The very important advantage of the research presented in this article is the application of a new type of reusable metal microelectrode array with an extended lifetime and simplicity of its surface preparation.

## Figures and Tables

**Table 1:** Analytical parameters characterizing the developed procedure for voltammetric thallium ions determination for 120 and 180 s of deposition.

Analytical Parameter	Value		Unit
	120 s	180 s	
Slope	3.95	7.14	nA/nmol L^−1^
Intercept	18.71	4.14	nA
R ^1^	0.9989	0.9988	-
Linear range	0.5–500	0.2–200	nmol L^−1^
LOD ^2^	0.22	0.08	nmol L^−1^

^1^ Correlation coefficient; ^2^ limit of detection.

**Table 2:** The comparison of parameters of analytical procedures of thallium ions determination using various mercury-free metal/metal film working electrodes using anodic stripping voltammetry.

Working Electrode	Linear Range [nmol L^−1^]	Detection Limit [nmol L^−1^]	Remarks	Ref.
BiFE	12–150	10.8	Rotating disc	[30]
BiFEs	0.05–5	0.021	Two BiFE electrodes	[31]
Bi-graphite electrode	49–4900	4.9	Mechanically renewed electrode	[32]
BiABE	0.5–49	0.005	-	[33]
GrE/Bi nanopowder	4.9–2446	0.15	Surface-modified electrode	[34]
BiF/SPE	5–1000	0.847	Integrated three-electrode sensor	[35]
	0.05–1	0.00671		
SPCE/MWCNTs/BiF	10–1000	2.8	-	[36]
BiµE	2–200	0.83	Solid metal microelectrode	[37]
SbFE	100–490	10	-	[41]
BiF/AuµE	0.5–500	0.22	-	[this work]
	0.2–200	0.08		

Abbreviations: BiABE—bismuth bulk annular band working electrode; GrE/Bi nanopowder—thick-film graphite electrode with Bi nanopowder; BiF/SPE—bismuth film screen-printed electrode; SPCE/MWCNTs/BiF—screen-printed carbon sensor modified with multiwalled carbon nanotubes and bismuth film; BiµE—bismuth microelectrode; SbFE—antimony film electrode; BiF/AuµE—solid gold microelectrode array modified with bismuth film.

**Table 3:** Relative Tl(I) analytical signal with and without the presence of interfering ions. Tl(I) concentration was 1 × 10^−8^ mol L^−1^.

Interfering Ions	Molar Excess of Interfering Ions	Relative Signal of Tl(I) (I/I_0_)
Cu(II)	20	0.82
	50	0.59
Pb(II)	50	0.96
	100	0.78
Fe(III)	100	0.95
	250	0.89
Zn(II)	100	0.92
	250	0.86
Cd(II)	100	0.98
	250	0.91
Mn(II)	100	1.03
	250	1.07

Abbreviations: I—Thallium peak current after the addition of an interfering ions excess; I_0_—thallium peak current before the addition of an interfering ions excess.

**Table 4:** The results of Tl(I) determination in certified reference material and real water sample.

Sample	Measured Value ± SD (n = 5) [μg L^−1^]	Certified Value ± SD [μg L^−1^]	Recovery [%]
CRM (TM 25.5)	29.0 ± 1.0	30.0 ± 2.8	96.7
**Sample**	**Added [nmol L^−1^]**	**Found** **± SD** **(n = 3) [nmol L^−1^]**	**Recovery [%]**
Bystrzyca River	0	0	-
	10.0	9.8 ± 0.2	98.0
	20.0	19.8 ± 0.4	99.0
	30.0	29.6 ± 0.6	98.7
	40.0	40.7 ± 1.1	101.8

## Data Availability

Data are contained within the article.

## Supplementary Materials

The following supporting information can be downloaded at: https://www.mdpi.com/article/10.3390/s24041206/s1, Figure S1: Anodic stripping voltammograms obtained for nine subsequent measurements performed from the same sample during repeatability studies. Tl(I) concentration was 1 × 10^−8^ mol L^−1^. Conditions of deposition: −1.25 V, 120 s.
