# Supplementary material for: Anodic Stripping Voltammetric Procedure of Thallium(I) Determination by Means of a Bismuth-Plated Gold-Based Microelectrode Array
Source: Sensors (Basel). 2024 Feb 13;24(4):1206. doi: 10.3390/s24041206 (PMC10892365; doi:10.3390/s24041206)
Supplement: Supplementary file 1 [file sensors-24-01206-s001.zip › sensors-2790238-supplementary.pdf]

Supplementary Materials

Anodic stripping voltammetric procedure of thallium(I) determination by means of a bismuth-plated gold-based microelectrode array

Mieczyslaw Korolczuk[1], Mateusz Ochab[1], Iwona Gęca[1,*]

[1] *Institute of Chemical Sciences, Faculty of Chemistry, Maria Curie Sklodowska University, 20-031 Lublin, Poland*

* e-mail: iwona.geca@mail.umcs.pl

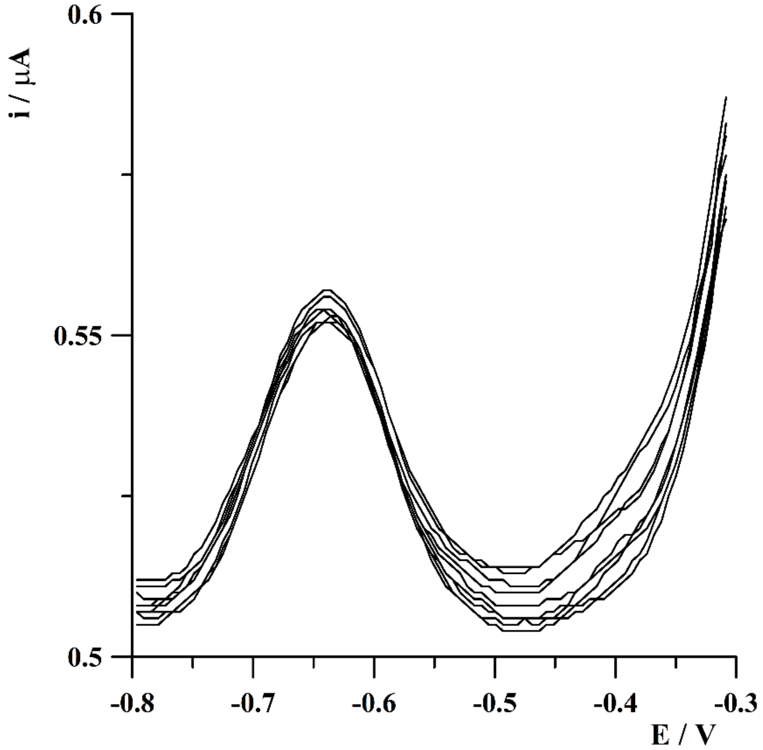

Figure S1. Anodic stripping voltammograms obtained for nine subsequent measurements performed from the same sample during repeatability studies. Tl(I) concentration was 1×10^{-8} mol L^{-1}. Conditions of deposition: -1.25 V, 120 s.
